# Persistent circulating unmetabolised folic acid in a setting of liberal voluntary folic acid fortification. Implications for further mandatory fortification?

**DOI:** 10.1186/1471-2458-9-295

**Published:** 2009-08-18

**Authors:** Mary R Sweeney, Anthony Staines, Leslie Daly, Aisling Traynor, Sean Daly, Steven W Bailey, Patricia B Alverson, June E Ayling, John M Scott

**Affiliations:** 1 UCD School of Public Health and Population Science, University College Dublin, Dublin 4, Ireland; 2 School of Nursing, Dublin City University, Glasnevin, Dublin 9, Ireland; 3 Coombe Women's and Infant's Hospital, Dublin 8, Ireland; 4 Department of Pharmacology, University of South Alabama, Mobile, Alabama 36688, USA; 5 Department of Biochemistry, University of Dublin, Trinity College, Dublin 2, Ireland

## Abstract

**Background:**

Ireland is an example of a country that has extensive voluntary fortification with folic acid. After a public consultation process, in 2006, the Food Safety Authority in Ireland FSAI [1] recommended mandatory fortification. However due to safety considerations this decision is now on hold. Before mandatory fortification goes ahead, existing levels of unmetabolised folic acid and their anticipated increase after fortification needs investigation because of the potential of folic acid to mask pernicious anaemia and possibly accelerate the growth of existing cancers. The aim of this study was to examine the levels of circulatory unmetabolised folic acid in Irish adults (both fasted and un-fasted) and new-born infants (fasted) before the proposed implementation of mandatory folic acid fortification. A secondary aim was to predict the increase in circulatory unmetabolised folic acid levels after fortification.

**Methods:**

*Study 1*. Setting: Irish Blood Transfusion Service (IBTS). Whole blood samples were collected from blood donors (n = 50) attending for routine blood donation sessions (representing the general population). Subjects were not fasted prior to sampling. *Study 2*. Setting: Coombe Women's and Infant's University Hospital, Dublin. Whole blood samples were collected by venipuncture from mothers (n = 20), and from their infant's umbilical-cords (n = 20) immediately after caesarean section. All women had been fasted for at least 8 hours prior to the surgery. A questionnaire on habitual and recent dietary intakes of folic acid was administered by an interviewer to all subjects. The data collection period was February to April 2006. Serum samples were analysed for plasma folate, plasma folic acid and red cell folate.

**Results:**

*Blood Donor Group*: Circulatory unmetabolised folic acid was present in 18 out of 20 mothers (fasted) (CI: 68.3%–99.8%) comprising 1.31% of total plasma folate, 17 out of 20 babies (fasted) (CI: 62.1%–96.8%), and 49 out of 50 blood donors (unfasted) (CI: 88.0%–99.9%), comprising 2.25% of total plasma folate,

**Conclusion:**

While the levels of circulatory unmetabolised folic acid reported are low, it is persistently present in women immediately after caesarean section who were fasting indicating that there would be a constant/habitual exposure of existing tumours to folic acid, with the potential for accelerated growth. Mandatory fortification might exacerbate this. This has implications for those with responsibility for drafting legislating in this area.

## Background

Folic acid and other micronutrients have been added on a "voluntary" basis to ready-to-eat breakfast cereals in the Republic of Ireland for over 15 years, to remedy perceived inadequate intake. Following the confirmation that supplementary folic acid could prevent ~72% of Neural Tube Defects [2] many foodstuffs fortified with folic acid, have been placed on the Irish market. Varying amounts of the pro-vitamin are added by food producers, to foods such as, milk, yoghurt, vegetable spreads, cereals, and breads.

In 2006 the FSAI conducted a public consultation process, after which it recommended folic acid fortification of most breads on sale in the Republic of Ireland at a level calculated to give an extra intake of 120 μg/day [1]. As part of their recommendations, consumers planning a pregnancy were encouraged to continue to take 400 μg/day of folic acid, in supplemental form. In addition, the existing range of "voluntary" fortified foods on sale would continue to be available. As we write, mandatory folic acid fortification in the Republic of Ireland has not yet been implemented and the most recent report published by the FSAI (March 2009) [3] has stated that mandatory fortification is now on hold until more evidence regarding its safety becomes available. In the UK mandatory folic acid fortification is also on-hold until an Independent Advisory Committee on Nutrition (SACN) [4] initiated by the Chief Medical Officer of England in October 2007 reviews the evidence in relation to safety.

These new safety concerns have arisen as a result of a Randomised Controlled Trial (RTC) which was conducted in the US. Two publications have now emerged from this RTC [5] and [6]. In the first paper [5] the authors showed that persons with a history of colorectal adenomas, consuming folic acid supplements as part of a randomized control trial, had an increased risk of more severe recurrence than a placebo group. The dose administered in the trial was 1 mg (the upper safe limit). The second paper [6] suggests that oral folic acid increases the risk of prostate cancer. These findings pose pressing public health concerns.

It has been known for some time that oral folic acid above certain threshold doses (~200 μg) results in the appearance of unmetabolised folic acid in serum as well as the normal metabolite 5-methyltetrahydrofolate [7]. Subsequent papers demonstrated a cumulative effect of circulatory unmetabolised folic acid from repeated consumption of folic acid [8], and circulatory unmetabolised folic acid [9] has now been found in foetal-cord blood, after passive consumption of 'voluntarily' fortified food by their mothers.

It is also known that folic acid intake can mask the early haematological manifestations of pernicious anaemia. This has been demonstrated both experimentally [10-13] and clinically [14-17]. However intakes of less than 1 mg a day in adults are not thought to have this masking effect.

In this study we set out to explore if circulatory unmetabolised folic is present in Irish people, living in the community, and exposed to the current range of "voluntarily" fortified foodstuffs, and if it persists even when fasting for some hours. In addition we wished to examine whether infants in utero would have unmetabolised folic acid in their cord-blood after a period of maternal fasting, as previous research has shown unmetabolised folic acid in cord-blood [9] but the mothers in that study had not been fasted at the time of sampling. A secondary aim of the study was to predict the increase in circulatory unmetabolised folic acid levels should a policy of mandatory fortification be introduced.

## Methods

### Blood collection and questionnaire administration

The data collection period was February to April 2006.

### Study 1 setting

Irish Blood Transfusion Service (IBTS), Dublin 8.

Blood donors attending for routine blood donation sessions were approached by a researcher on entry to the clinic and informed consent was obtained. Whole blood samples were collected from blood donors (n = 50) (males 42, females 8, age range 27–60 years). Subjects had consumed their normal diet prior to the sampling.

### Study 2 setting

Coombe Women's and Infant's Hospital, Dublin 8.

Mothers about to undergo routine caesarean section were approached by the researcher and informed consent was obtained. Whole blood samples were collected by venipucture from mothers (n = 20) (age range 26–39 years) and the umbilical cords of their babies (n = 20) (on the placental side), immediately after caesarean section. All the women had been routinely fasted for at least 8 hours prior to the surgery. None of the pregnant women in this study were consuming folic acid supplements at the time of this study. This was validated by our dietary questionnaire which revealed that the most recent consumption of folic acid supplements by any woman was 120 days prior to the blood sampling for this study.

### Questionnaire data

A questionnaire intended to assess habitual and recent dietary intakes of folic acid was administered by an interviewer to all adult subjects. This had sections covering the main dietary sources of folic acid supplements and fortified foods available in Ireland. A comprehensive database of all these products was constructed to facilitate analysis of the questionnaires. In addition their general health was documented. Any persons with a metabolic, liver, renal or chronic gastrointestinal condition were excluded.

### Laboratory analysis

Whole blood was collected into 3 ml EDTA tubes and stored in light-proof containers until transfer to the laboratory. Plasma was separated from the whole blood by centrifugation at 2500 for 12 minutes. Samples were separated into 1.5 ml tubes and stored at -20°C until batch analysis. Plasma folate and red cell folate analysis were conducted by the *L. casei *microbiological assay described by Molloy & Scott (1997) [18]. Unmetabolised folic acid was measured by a column switching HPLC method [[19], Appendix 1].

### Statistical analysis

Statistical analysis was based on the Wilcoxon signed rank test and the calculation of exact 95% confidence limits for proportions. The computer package SPSS V.11 was used to perform regression analysis. Using a regression equation as per Quinlivan & Gregory (2003) [20] we estimated the average increase in plasma folate in the population, that might occur as a result of mandatory fortification, and we estimated the impact of this on population circulatory unmetabolised folic acid levels. This calculation makes the assumption that unmetabolised folic acid will increase linearly as total plasma folate increases.

### Ethical approval

After permission from the Medical Director of the IBTS, ethical approval was obtained from University College Dublin Ethic's Committee and the Coombe Women's and Infant's Hospital, Dublin 8.

## Results

### Mother and baby group

**Figure 1:**
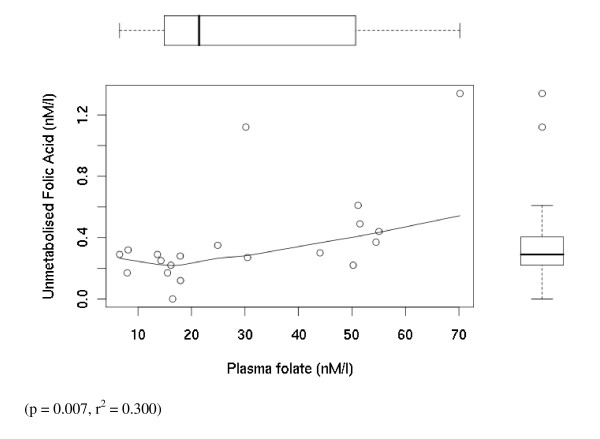
**Individual level data for the Mother's (fasted group) showing unmetabolised folic acid nM/L plotted against plasma total folate nM/l**. The box plots on the x and y axis respectively indicate the univariate distribution of plasma folate and plasma unmetabolised folic acid. In each box plot the line across the box indicates the median point, the whiskers indicate the range of the data, and the 25 and 75% centiles are indicated by the box itself.

Folic acid was present in 17 out of 20 babies (CI: 62.1%–96.8%) and 18 out of 20 mothers (fasted) (CI: 68.3%–99.8%) (Table 1 & Figure 1). There was a significant correlation between the maternal plasma folate concentrations and maternal plasma unmetabolised folic acid concentrations (p = 0.007, r^2 ^= 0.300), and maternal habitual folic acid intakes were correlated with maternal plasma folate concentrations (p = 0.001). Additionally we found a significant correlation between the maternal folic acid concentrations and cord blood folic acid concentrations (p = 0.004, r^2 ^= 0.378).

**Table 1:** Fasted Group.

Mothers	RCF (nM/L)	PFOL (nM/L)	PFA (nM/L)	% PFA/PFOL	FA intake daily (μg)	Cord-sample	PFA (nM/L)
Mean	1063.10	29.83	0.39	1.31	404.35	Mean	0.27
S.D.	506.15	19.57	0.325		310.77	S.D.	0.15
Median	945.00	21.42	0.290		297.5	Median	0.28
Minimum	278.00	6.56	0.00		82.0	Minimum	0.00
Maximum	2180.00	70.18	1.34		1321.00	Maximum	0.60

Mean red cell folate, plasma total folate and unmetabolised folic acid concentrations in the mothers (n = 20) and cord-blood group (n = 20).

RCF – Red cell folate

PFOL – Plasma total folate

PFA – Plasma folic acid

%PFA/PFOL – percentage of plasma folic acid to plasma folate

Cord-blood total folate analysis was not possible due to insufficient sample.

### Blood donor group

**Figure 2:**
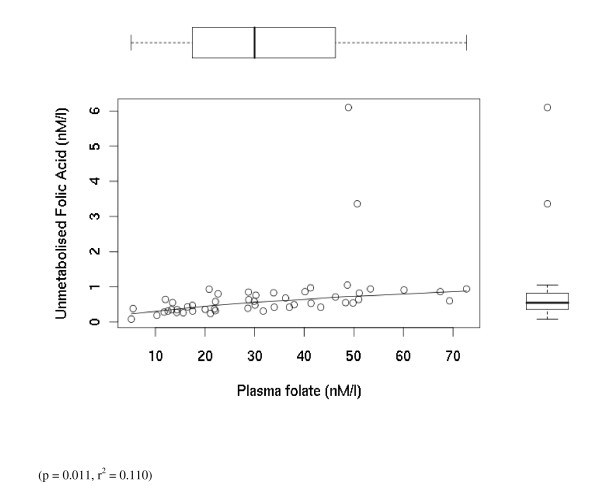
**Individual level data for the blood donor's (unfasted group) showing unmetabolised folic acid nM/L plotted against plasma total folate nM/l**. Outliers are included in this figure. The box plots on the x and y axis respectively indicate the univariate distribution of plasma folate and plasma unmetabolised folic acid. In each box plot the line across the box indicates the median point, the whiskers indicate the range of the data, and the 25 and 75% centiles are indicated by the box itself. the first. At this lower voltage specifically the folic acid peak decreases by about 95%, thus permitting not only its definitive identification, but also facilitation of placement of baselines for quantification of peak heights using an Empower (Waters) data system. The limit of detection (S/N = 3) was 0.15 nM in plasma, and the intra assay CV = 3%.

Unmetabolised folic acid was present in 49 out of 50 blood donors (unfasted) (CI: 88.0%–99.9%) (Table 2 and 3, & Figure 2). After removing 2 samples, which were outliers, with very high unmetabolised folic acid levels relative to the others sampled, we examined inter-relationships between plasma unmetabolised folic acid, plasma folate, red cell folate, habitual folic acid and recent folic acid intakes using regression techniques. Our results show that habitual folic acid intakes are significantly correlated with plasma folate levels (p = 0.009 r^2 ^= 0.115). In addition we find that plasma folate is related to plasma unmetabolised folic acid concentrations (p = 0.011, r2 = 0.110).

**Table 2:** Unfasted group with outliers included.

	RCF (nmol/L)	PFOL (nmol/L)	PFA (nmol/L)	% PFA/PFOL	FA intake daily (μg)	FA intake in past 24 hours (μg)
Mean S.D.	1034.56 349.07	32.02 17.25	0.722 0.903	2.25	196.85 208.97	122.60 191.49
Median	1074.50	29.99	0.545		124.37	68.00
Minimum	356.00	5.13	0.08		0.00	0.00
Maximum	1778.0	72.71	6.10		962.30	1150

Mean red cell folate, plasma total folate and unmetabolised folic acid concentrations in the blood donor group (n = 50).

RCF – Red cell folate

PFOL – Plasma total folate

PFA – Plasma folic acid

%PFA/PFOL – percentage of plasma folic acid to plasma folate

In table 2(a) two high outliers included for the purpose of statistical analysis are included in this table.

FA intake daily (μg) – reflects typical daily folic acid intake from supplements and fortified foodstuffs.

FA intake in past 24 hours (μg) – reflects folic acid intake in the previous 24 hours from supplements and fortified foodstuffs.

**Table 3:** Unfasted group with outliers excluded.

	RCF (nmol/L)	PFOL (nmol/L)	PFA (nmol/L)	% PFA/PFOL	FA intake daily (μg)	FA intake in past 24 hours (μg)
Mean S.D.	1029.02 355.142	31.28 17.21	0.56 0.24	1.79	182.84 199.85	94.63 112.79
Median	1063.50	29.36	0.54		117.07	45.00
Minimum	356.00	5.13	0.08		0.00	0.00
Maximum	1778.00	72.71	1.05		962.30	500.00

Mean red cell folate, plasma total folate and unmetabolised folic acid concentrations in the blood donor group (n = 50).

RCF – Red cell folate

PFOL – Plasma total folate

PFA – Plasma folic acid

%PFA/PFOL – percentage of plasma folic acid to plasma folate

In table 2(b) two high outliers excluded for the purpose of statistical analysis are also excluded in this table.

FA intake daily (μg) – reflects typical daily folic acid intake from supplements and fortified foodstuffs.

FA intake in past 24 hours (μg) – reflects folic acid intake in the previous 24 hours from supplements and fortified foodstuffs.

**Footnote: **FA intakes daily and habitually show marked variability. This can be explained as follows. In attempting to capture habitual intakes a questionnaire similar to the food frequency questionnaire (which is a validated tool) was used. This questionnaire captures habitual intakes by documenting how much of the "nutrient of interest" is consumed per serving and typically on how days per week, month and then per year. The total amount is then divided by 365 to give a typical daily intake. Recent intakes are more easily captured as memory recall is not an issue and the intake data do not have to account for variability between days, weeks and months.

Using a regression equation as per Quinlivan & Gregory (2003) [20] (based on change in serum folate = 1.0254 + 0.0514 X), relating change in plasma folate levels to an increase in folic acid intakes per day together with the estimated folic acid intakes likely to be achieved with the proposed fortification in Ireland (~120 μg/day), we estimated the average increase in plasma folate in the population to be 3.1 μg/L. The mean level of plasma folate currently in the general population of Ireland (based on our sample of blood donors) was 13.81 μg/L. Thus, following fortification, we would expect a mean plasma folate increase of 3.1 μg/L, giving a final value of 16.9 μg/L. Hence putting our equation relating unmetabolised folic acid to absolute levels of plasma folate we predict a population level of 0.623 μg/L for unmetabolised folic acid levels, i.e. approximately 12% increase from current levels.

## Discussion

The aim of sampling the unfasted group was to see what levels of unmetabolised folic acid are present in an Irish population randomly sampled (not fasted). We did not wish to capture basal levels in this population hence the group was not fasted. In this study, we have shown that circulatory unmetabolised folic acid is present in the majority of our sample, regardless of whether they are fasted or not, comprising 2.25% of total plasma folate in an unfasted group, and 1.31% of total plasma folate in a fasted group. This implies constant exposure of both normal cells, and potential tumour cells, to this pro-vitamin amongst Irish consumers. In terms of the predicted increase that will arise if mandatory fortification goes ahead, we predict the increase to be in the region of 12%, A recent US study [21] which assayed samples from fasting subjects before and after fortification has shown that the proportion of plasma folic acid to plasma folate in non-supplement users increased from 9% to 19.1% after fortification and in supplement users there was an increase from 15.9% to 24.3%.

The upper safe limit for folic acid intake is not known definitively, but has been traditionally considered to be in the region of 1 mg/day, based on what we know about pernicious anaemia masking. There is currently no information available to allow us determine what constitutes a safe level of intake of folic acid if cancer acceleration is the criterion under consideration. Furthermore we do not know whether it is the unmetabolised folic acid or a general increase in folate status which may be responsible for any potential cancer acceleration effects. While 1 mg/day was shown to accelerate tumour growth in the Cole (2007) [5] and Figueiredo (2009) [6] studies, we have no way of knowing if these effects would have occurred at intakes lower than this.

The magnitude of increase in circulatory unmetabolised folic acid from 1 mg of folic acid administered orally, has only been investigated in one previous published study [9], This study examined the effect on circulatory unmetabolised folic acid of 1 mg of folic acid in fortified bread in four subjects from baseline to 4 hours post-prandially. These subjects were not pre-saturated with folic acid supplements, and neither were they regular users of supplements. The proportion of unmetabolised folic acid to total serum folate at T-max was 3.9%.

It is worth noting in this discussion that factors other than folic acid intake, such as an individual's capacity to absorb, or metabolise folic acid, may also be an important consideration in establishing safe intakes/concentrations. Proven variation in di-hydrofolate reductase activity may require further exploration [22].

A limitation of the study is that samples from fasting males and non-pregnant females were not included in this study.

## Conclusion

In conclusion the majority of the population in Ireland, now exposed to the liberal voluntary fortification regime, appears to have circulating unmetabolised folic acid, as measured in plasma from non-fasting subjects and fasting women who have undergone caesarean section. The extra folic acid to which consumers will be exposed if mandatory folic acid fortification goes ahead will undoubtedly increase these levels further. The consequences of this are unknown but should continue to be of concern for those with responsibility for drafting legislation in this area.

## Competing interests

Authors Steven W. Bailey and June E. Ayling are co-inventors of patents in the folate field but the compounds which are the subject of these patents are not examined in this manuscript. The authors declare that they have no other competing interests. The lead authors had full access to all the data in the study and had final responsibility for the decision to submit for publication.

## Authors' contributions

MRS had the original idea for the study. She designed the study, obtained the ethical approval, supervised the field-work, inputted the data, performed the statistical analysis, and drafted the paper. AS provided advice on the study design and was involved in drafting the manuscript and revising it for intellectual content. LD advised on the study design and statistical analysis and was involved in drafting the manuscript and revising it for intellectual content. AT conducted the dietary interviews. SD facilitated the collection of bloods from the Coombe Women's and Infant's Hospital and was involved in revising the manuscript for intellectual content. JA and SB provided advice on the study design and were involved in drafting the manuscript and revising it for intellectual content. JA's laboratory performed the unmetabolised folic acid analysis. PA performed the unmetabolised folic acid analyses. JS provided advice on the study design and was involved in drafting the manuscript and revising it for intellectual content. JS's laboratory conducted the folate measurements. All authors read and approved the final manuscript.

## Appendix 1

### Assay for unmetabolised folic acid

Briefly, to 360 μL EDTA plasma in a microfuge tube was added 180 μL distilled water, and after mixing and closure with a screw cap boiled for 30 min. After cooling on ice, the tubes were fast frozen in liquid nitrogen and thawed quickly in an ambient temperature water bath to allow for greater compaction of the pellet during subsequent centrifugation at 25,000 × g for 15 min without cooling. The supernatant was then removed, its volume measured, 0.1 volumes of 6 M trichloroacetic acid added with mixing, and then centrifuged for 25,000 × g for 10 min at 4°C. The supernatant was stored at -80°C until analysis. Standards were generated by this same procedure after spiking pooled plasma with a known amount of folic acid (determined using ε_281 nm _= 27,600 at pH 7.0). The level of the standard was corrected for the measured value of endogenous unmetabolized folic acid in the pooled plasma prior to spiking. The use of spiked plasma not only takes into account the recovery during workup (~87%), but also prevents adsorption of folic acid to the walls of the autosampler vials during analysis.

For the first stage of the HPLC analysis 50 μL of sample or standard was injected with a Varian ProStar 420 autosampler into a Zorbax C8-SB column (1.8 μ, 50 × 4.6 mm, Agilent) protected with a Hypersil C8 BDS (3 μ, 4.0 × 3.0 mm SecurityGuard cartridge, Phenomenex), both placed in a water jacket maintained at 40°C. This was eluted at 0.75 ml/min with 20 mM ammonium hydroxide plus acetic acid to pH 3.6/acetonitrile (93:7). Just prior to the elution of the folic acid from the above Zorbax -C8 column, the mobile phase was automatically redirected by a switching valve (Vici-Cheminert model C72) from waste to the head of two coupled Luna phenyl-hexyl columns (3 μ, each 150 × 4.6, Phenomenex) placed in a water jacket maintained at 40°C. After complete transfer of the folic acid peak from the Zorbax-C8 to the phenyl-hexyl columns, the switching valve was returned to the initial state causing the remainder of the eluate from the first column to again go to waste. The phenyl-hexyl columns were developed with 40 mM ammonium hydroxide plus phosphoric acid to pH 2.1/acetonitrile (9:1) at a flow of 0.8 ml/min. The pressures of the two pumps were equalized by attaching the appropriate length of 65 μ PEEK capillary tubing (Upchurch) to the waste port of the switching valve. The timing of elution from the Zorbax-C8 column was determined by connecting this capillary directly to a photodiode array (Waters model 996) and injection of 50 μL of 400 nm folic acid in 0.55 M trichloroacetic acid without switching. The above combination of columns and mobile phases was specially optimized for the more challenging chromatography of non-fasted samples.

Fluorescence detection of folic acid was greatly enhanced by coulometric electrochemical oxidation of the mobile phase from the phenyl-hexyl columns by passage first through an ESA 1010 dual cell with both electrodes set to the potential of maximal response (ESA model 5100A controller, typically between 0.45 V and 0.6 V). The output of the electrochemical cell, primarily the folic acid cleavage products pterin-6-carboxylic acid and p-aminobenzoyl glutamate, was fed into a Waters model 2476 fluorometer set to 362 nm excitation and 455 nm emission. After analysis of the spiked plasma standards, 50 μL of 0.55 M trichloroacetic acid was injected to establish that the system was free of residual folic acid. In the event of detection of a folic acid peak, several injections of 0.1 M Tris-Cl, pH 8.0/acetonitrile (1:1) were made without switching, to clear the autosampler of any carryover. All samples were injected twice (from a cooled and dark sample tray), the second time at an oxidation potential 0.3 V lower than the first. At this lower voltage specifically the folic acid peak decreases by about 95%, thus permitting not only its definitive identification, but also facilitation of placement of baselines for quantification of peak heights using an Empower (Waters) data system. The limit of detection (S/N = 3) was 0.15 nM in plasma, and the intra assay CV = 3%.

## Pre-publication history

The pre-publication history for this paper can be accessed here:



## References

[B1] Food Safety Authority of Ireland Consultation Committee. http://www.fsai.ie.

[B2] MRC Vitamin Research Group (1991). Prevention of neural tube defects: results of the Medical Research Council Study. Lancet.

[B3] Food Safety Authority of Ireland (2009). Report of the implementation Group on Folic Acid Food Fortification to the Department of Health and Children. Department of Health and Children.

[B4] Independent Advisory Committee on Nutrition (SACN). http://www.sacn.gov.uk.

[B5] Cole BF, Baron JA, Sandler RS, Haile RW, Ahnen DJ, Bresalier RS, McKeown-Eyssen G, Summers RW, Rothstein RI, Burke CA, Snover DC, Church TR, Allen JI, Robertson DJ, Beck GJ, Bond JH, Byers T, Mandel JS, Mott LA, Pearson LH, Barry EL, Rees JR, Marcon N, Saibil F, Ueland PM, Greenberg ER (2007). Folic acid for the prevention of colorectal adenomas: a randomized clinical trial. Journal of the American Medical Association.

[B6] Figueiredo JC, Grau MV, Haile RW, Sandler RS, Summers RW, Bresalier RS, Burke CA, McKeown-Eyssen GE, Baron JA (2009). Folic acid and risk of prostate cancer: results from a randomized clinical trial. Journal of the National Cancer Institute.

[B7] Kelly P, Mc Partlin J, Scott JM (1996). A combined High-Performance liquid chromatographic microbiological assay for serum unmetabolised folic acid. Analytical Biochemistry.

[B8] Sweeney MR, McPartlin J, Weir DG, Leslie D, Scott JM (2006). Post-prandial serum folic acid response to multiple doses of folic acid in fortified bread. British Journal of Nutrition.

[B9] Sweeney MR, McPartlin J, Weir DJ, Daly S, Pentieva K, Daly L, Scott JM (2005). Evidence of unmetabolised folic acid in cord-blood of new-born and serum of four-day old infants. British Journal of Nutrition.

[B10] Tisman G, Herbert V (1973). B-12 dependence of cell uptake of serum folate: explanation for high serum folate and cell folate depletion in B-12 deficiency. Blood.

[B11] Metz J, Kelly A, Swett VC, Waxman S, Herbert V (1968). Deranged DNA synthesis by bone marrow from vitamins B-12 deficient humans. British Journal of Haematology.

[B12] Zittoun J, Marquet J, Zittoun R (1978). Effect of folate and cobalamin compounds on the deoxyuridine supression test in vitamin B-12 and folate deficiency. Blood.

[B13] Ganeshaguru K, Hoffbrand AV (1978). The effect of deoxyuridine, vitamin B-12, folate and alcohol on the uptake of thymidine and on the deoxynucleoside triphosphate concentrations in normal and megaloblastic cells. British Journal of Haematology.

[B14] Heinle RM, Welch AD (1974). Folic acid in pernicious anaemia. Failure to prevent neurologic relapse. Journal of the American Medical Association.

[B15] Bethal FH, Stergis CC (1948). The relation of therapy in pernicious anaemia to changes in the nervous system. Early and late results in a series of cases observed for periods of not less than ten years, and early results of treatment with folic acid. Blood.

[B16] Vilter RW, Horrigan D, Mueller JF, Mueller TJ, Vilter CF, Hawkins V, Seaman A (1950). Studies on the relationships of vitamin B12, folic acid, thymine, uracil, and methyl group donors in persons with pernicious anaemia and related megaloblastic anaemias. Blood.

[B17] Israels MCG, Wilkinson JF (1949). Risk of neurological complications in pernicious anaemia treated with folic acid. British Medical Journal.

[B18] Molloy A, Scott JM (1997). Microbiological assay for serum, plasma and red cell folate using cryopreserved microtitre plate method. Methods in Enzymology.

[B19] Bailey SW, Syslo MC, Ayling JE (2002). A HPLC Method for Analysis of Unreduced Folic Acid in Blood. Federation of American Societies for Experimental Biology J.

[B20] Quinlivan EP, Gregory JF (2003). Effect of food fortification on folic acid intake in the United States. American Journal of Clinical Nutrition.

[B21] Kalmbach RD, Choumenkovitch SF, Troen AM, D'Agostino R, Jacques PF, Selhub J (2008). Circulating folic acid in plasma: relation to folic acid fortification. Am J Clin Nutr.

[B22] Bailey SW, Ayling JE The Extremely Slow and Variable Activity of Dihydrofolate Reductase in Human Liver and its Implications for High Folic Acid Intake. Proc Natl Acad Sci USA.

